# Adverse Childhood Experiences (ACEs) and Adiposity in Adolescents: A Cross‐Cohort Comparison

**DOI:** 10.1002/oby.22035

**Published:** 2017-11-14

**Authors:** Ana Luiza G. Soares, Alicia Matijasevich, Ana M.B. Menezes, Maria Cecília Assunção, Fernando C. Wehrmeister, Laura D. Howe, Helen Gonçalves

**Affiliations:** ^1^ Postgraduate Program in Epidemiology, Federal University of Pelotas Pelotas Brazil; ^2^ MRC Integrative Epidemiology Unit Population Health Sciences, Bristol Medical School, University of Bristol Bristol UK; ^3^ Department of Preventive Medicine School of Medicine, University of São Paulo São Paulo Brazil

## Abstract

**Objective:**

This study aimed to assess the association between adverse childhood experiences (ACEs) and adiposity in adolescents from two cohorts in different socioeconomic contexts.

**Methods:**

Data from the Avon Longitudinal Study of Parents and Children (ALSPAC, United Kingdom) and the 1993 Pelotas Cohort (Brazil) were used. Six ACEs were assessed in both cohorts up to age 15. At 15 years, body mass index (BMI) and waist circumference (WC) were measured, and at 18 years, BMI, fat mass index, and android fat percentage were assessed.

**Results:**

Few associations were observed between ACEs and adiposity at 15 years, and they were not consistent across cohorts. For adiposity at age 15 in ALSPAC, physical abuse had a positive association with WC, and domestic violence had a positive association with both WC and BMI. A dose‐response relationship between the ACE score and both WC and BMI at 15 years was observed in ALSPAC. In the 1993 Pelotas Cohort, the associations found in crude analysis were no longer evident after adjustment.

**Conclusions:**

This study found some evidence of an association between an ACE score and adiposity in adolescence in a United Kingdom cohort but no evidence of association in a Brazilian cohort. Residual confounding or context‐specific relationships could explain the different pattern of associations.

## Introduction

Adverse childhood experiences (ACEs) have been associated with several lifelong consequences, including poor psychological and physical health outcomes 1, 2. Psychiatric disorders and health‐related risk behaviors, such as smoking, alcohol use, and risky sexual behavior, as well as other health outcomes, such as high blood pressure, overweight or obesity, cardiovascular disease, diabetes, and cancer, are some of the outcomes shown to be associated with the occurrence of ACEs 2, 3, 4, 5. Physical abuse, sexual abuse, neglect, and interpersonal violence are the ACEs more frequently assessed, and the co‐occurrence of multiple types of ACEs is common and has to be considered 6.

There is clear evidence of an association between ACEs and adiposity measures in adult populations. Meta‐analyses have shown an increased risk of obesity in adults who experienced ACEs, and the results were robust and similar across the studies, with pooled odds ratios varying from 1.23 to 1.34 7, 8. However, when analyzing studies carried out with children and adolescents, evidence of association was inconclusive in this age group (pooled odds ratio = 1.13; 95% CI: 0.92‐1.39) 7.

Elevated body mass index (BMI) in childhood and adolescence has been associated with several obesity‐related morbidities in adult life, such as diabetes, coronary heart disease, and some types of cancer 9. The identification of the association between ACEs and adiposity in adolescence is therefore important to plan early interventions to alleviate the health effects of ACEs. Even though BMI is a good indicator of adiposity, it does not reflect fat distribution, which is important for cardiometabolic disease risk 10. To date, no study has assessed the association of ACEs and more detailed measures of adiposity, such as body fat quantity and distribution, in adolescence. Most of the evidence of the relationship between ACEs and BMI comes from high‐income countries, and more studies are needed in low‐ and middle‐income countries considering the prevalence and socioeconomic distribution of ACEs differ between settings 6.

Thus, this study aimed to assess the association of several individual ACEs as well as an ACE score with adiposity measures (BMI, waist circumference [WC], fat mass index [FMI], and android fat percentage) in adolescents from two birth cohorts within contrasting socioeconomic contexts: the United Kingdom and Brazil. This cross‐cohort comparison design is a useful tool to explore potential residual socioeconomic confounding in the association of interest 11. This approach is based on the notion that if an observed association is causal, it should be evident in both cohorts, despite the different confounding structures 11.

## Methods

### Research settings

Data from the Avon Longitudinal Study of Parents and Children (ALSPAC, United Kingdom) and the 1993 Pelotas Birth Cohort (Brazil) were used.

ALSPAC recruited pregnant women resident in the Avon area of the United Kingdom with an expected delivery date between April 1, 1991, and December 31, 1992. Since then, mothers, partners, and children have been followed up regularly through questionnaires and clinical assessments 12. The child cohort consists of 14,775 live‐born children (75.7% of the eligible live births). At the 15‐year clinic assessment (mean age: 15.5 years), 5,509 children participated (follow‐up rate: 37%), and at 18 years (mean age: 17.8 years), 5,196 adolescents attended the clinic (follow‐up rate: 35.2%) 12. The ALSPAC study website contains details of all the data that are available through a fully searchable data dictionary (http://www.bris.ac.uk/alspac/researchers/data-access/data-dictionary/).

The 1993 Pelotas Birth Cohort recruited all children born alive in hospitals in the urban area of the city of Pelotas, Southern Brazil, between January 1, 1993, and December 31, 1993 13. During this period, 5,265 births were recorded and 5,249 agreed to take part in the study. Since then, the full cohort or subsamples have been followed. At 15 years (mean age: 14.7 years), the follow‐up rate was 85.7% (*n* = 4,325), and at 18 years (mean age: 18.4 years), 4,106 were interviewed (follow‐up rate: 81.3%) 14. The questionnaires used for the 1993 Pelotas Birth Cohort are available at http://www.epidemio-ufpel.org.br/site/content/coorte_1993/.

### Measures

#### Exposure

The selection of the ACEs included in our analysis was based on the ACE Study 1. The following six ACEs were assessed in both cohorts: physical abuse, sexual abuse, domestic violence, parental separation or divorce, separation from parents (being separated from the parents), and maternal mental health. Additional ACEs were also assessed in only one of the two cohorts: parental alcohol or drug problems were assessed in ALSPAC, and parental death, physical neglect, and emotional neglect were assessed in the 1993 Pelotas Cohort. In ALSPAC, all the ACEs were mother‐reported, and in the 1993 Pelotas Cohort, most of them were self‐reported in adolescence. Details of how the ACEs were assessed in ALSPAC and in the 1993 Pelotas Cohort are presented in Table 1.

**Table 1:** Questions used to assess adverse childhood experiences (ACEs) and time point of assessment in each cohort: ALSPAC study, United Kingdom, and 1993 Pelotas Cohort, Brazil

ACE	Question used	Time point of assessment
**Physical abuse**	**1993 Pelotas Cohort**: Has an adult of your family or someone who was looking after you hit you in a way that left you hurt or bruised?	15 y
	**ALSPAC**: Since she was […] old, she was physically hurt by someone, or Since […] your partner was physically cruel to your children, or Since […] you were physically cruel to your children	2, 3, 4, 5, 6, 7, and 9 y 8 mo, 2, 3, 4, 5, 6, 9, and 11 y
**Sexual abuse**	**1993 Pelotas Cohort**: Has anyone ever tried to do sexual things to you against your will, threatening or hurting you?	15 y
	**ALSPAC**: Since she was […] old, was she sexually abused	2, 3, 4, 5, 6, 7, and 9 y
**Domestic violence**	**1993 Pelotas Cohort**: Have there ever been fights with physical assault in your household between adults, or has an adult ever assaulted a child or adolescent?	15 y
	**ALSPAC**: Since […] your partner was physically cruel to you	2, 3, 4, 5, 6, 9, and 11 y
**Parental separation**	**1993 Pelotas Cohort**: Does the < name>'s natural father live in this house? / Do you have a husband who lives here? or Are your parents divorced?	11 y 15 y
	**ALSPAC**: Since […] you were divorced or Did this happen to you since you were aged […]: parents divorced or separated	8 mo, 2, 3, 4, 5, 6, 7, 8, 9, and 11 y 16 y
**Separation from parents**	**1993 Pelotas Cohort**: Have you ever been separated from your parents to be looked after by someone else?	15 y
	**ALSPAC**: Child was separated from his/her mother since his/her […] birthday or Child was separated from his/her father since his/her […] birthday	2, 3, 4, 5, 6, 7, and 9 y
**Maternal mental health problem**	**1993 Pelotas Cohort**: Self‐reported questionnaire (SRQ‐20)	11 y
	**ALSPAC**: Edinburgh Postnatal Depression Scale (EPDS)	2 y
**Parental death**	**1993 Pelotas Cohort**: Is < name>'s natural mother/father still alive? or Are your mother/father still alive or has she/he died?	11 and 15 y
**Physical neglect**	**1993 Pelotas Cohort**: Have you ever not had enough food at home or had to wear dirty or worn clothes because you had no others?	15 y
**Emotional neglect**	**1993 Pelotas Cohort**: Have you ever thought or felt that your parents did not want you to have been born? or Have you ever thought or felt that someone of your family hates you?	15 y
**Parental alcohol or drug problems**	**ALSPAC**: Have you ever had alcoholism? or How often have you drunk alcoholic drinks before this pregnancy/before your partner became pregnant? (> 2 glasses/d) or Have you ever had drug addiction? or How often did you smoke marijuana/grass/cannabis/ganja in the 6 months before you conceived or before your partner conceived? (any frequency reported)	12 wk 18 wk 12 wk 18 wk

A score of cumulative ACE exposure was generated by using the six ACEs available for both cohorts. The ACE score varied from 0 (no experience of ACE) to 6 (exposed to all ACEs). Sensitivity analyses were carried out in both cohorts by adding to the score the additional ACEs available in each cohort, and so this second score could take values from 0 to 7 in ALSPAC and from 0 to 9 in the 1993 Pelotas Cohort.

#### Outcomes

BMI was evaluated at ages 15 and 18 in both cohorts, WC was measured at age 15, and FMI and android fat percentage were assessed at 18 years. BMI was calculated by dividing weight (kilograms) by height (meters squared), both measured at the clinic visit. WC was measured at the midpoint between the lower ribs and the iliac crest in ALSPAC and at the narrowest point of the waist in the 1993 Pelotas Cohort. FMI was calculated by dividing total fat mass (kilograms), measured from dual‐energy x‐ray absorptiometry, by height (meters squared), and android fat percentage was calculated as a proportion of android fat mass (kilograms) to total body fat mass (kilograms).

Full details of how these variables were assessed are provided in the online Supporting Information.

#### Covariates

Family income, maternal education or schooling, maternal age, maternal smoking status at pregnancy, maternal prepregnancy BMI, birth weight, ethnicity (ALSPAC), skin color (1993 Pelotas Cohort), and gender were assessed in both cohorts.

Full details of how these variables were assessed are provided in the online Supporting Information.

### Statistical analysis

The analysis was restricted to those individuals with complete data on all ACEs (7 ACEs in ALSPAC and 9 ACEs in the 1993 Pelotas Cohort) and at least one of the outcomes (*N* = 4,444 for ALSPAC and 3,924 for the 1993 Pelotas Cohort). In order to increase efficiency in the analysis and minimize selection bias, multiple imputation by chained equations was used in both cohorts. The imputation equations included all ACEs, outcomes, and covariates and were stratified by sex. Logarithm transformation was used when continuous variables were not normally distributed. Logistic, ordinal logistic, and linear regression models were used as appropriate, and 20 cycles of regression switching were derived. The comparison of the distribution of imputed variables in the imputed data sets and the observed data (with no imputation) for ALSPAC and 1993 Pelotas Cohort is shown in Supporting Information Tables S1‐S2. The distributions in imputed data sets were similar to those in the observed data sets. All analyses were carried out using imputed data by combining estimates using Rubin's rule.

Descriptive statistics were generated for both cohorts, and the distributions of socioeconomic and demographic characteristics, ACEs, and adiposity measures were explored, comparing those participants included and not included in the analyses because of loss to follow‐up or missing data. The prevalence of each ACE and of the ACE score was also described according to gender in both cohorts (presented in the Supporting Information).

Unadjusted linear regression analyses were carried out, examining associations of individual ACEs and the ACE score with adiposity measures. We then adjusted the analyses for the covariates defined above. Gender‐stratified analyses were also carried out as it is possible that the health consequences of ACEs differ by gender. Because this was an exploratory analysis, the gender‐stratified results are presented in the Supporting Information.

In order to explore residual confounding by socioeconomic status (SES), associations of family income with individual ACEs, the ACE score, and adiposity measures were assessed in both cohorts.

The analyses were performed in Stata software version 14.1 (Stata Corp., College Station, Texas).

### Ethical approval

Ethical approval for the ALSPAC study was obtained from the ALSPAC Law and Ethics Committee and the Local Research Ethics Committee. The study protocol of the 1993 Pelotas Cohort was approved by the Medical Ethics Committee of the Federal University of Pelotas, affiliated with the Brazilian Federal Medical Council.

## Results

The characteristics of the participants included and not included in the analysis because of missing data or loss to follow‐up in both cohorts are presented in Table 2. In ALSPAC, those excluded from the analysis because of missing data or loss to follow‐up had a lower prevalence of physical abuse, parental separation, and separation from parents, a higher prevalence of domestic violence and maternal mental health problems, and a higher ACE score (Table 2). In the 1993 Pelotas Cohort, those not included in the analysis had a higher prevalence of separation from parents and parental death. In ALSPAC, those excluded from the analysis had a higher BMI and a higher FMI than those included in the analysis, while in the 1993 Pelotas Cohort, those excluded had a lower FMI and higher android fat at age 18. In both cohorts, participants excluded from the analysis were more likely to be male and to have lower family income, maternal schooling or education, and birth weight (Supporting Information Table S3). In ALSPAC, those not included were also more likely to report nonwhite ethnicity and to have lower maternal age, smoking mothers, and mothers with both underweight and excessive weight. In the 1993 Pelotas Cohort, those excluded were more likely to have mothers with a lower BMI.

**Table 2:** Description of the ACEs and adiposity measures of participants with complete data compared with participants with missing data or lost to follow‐up: ALSPAC study, United Kingdom, and 1993 Pelotas Cohort, Brazil

	ALSPAC			1993 Pelotas Cohort		
	Participants included in the analysis	Participants excluded from the analysis	*P* value^a^	Participants included in the analysis	Participants excluded from the analysis	*P* value^a^
***ACEs***						
**Physical abuse**	*n* = 4,444	*n* = 7,848	0.001	*n* = 3,924	*n* = 165	0.458
	4.8%	3.5%		7.0 %	8.3%	
**Sexual abuse**	*n* = 4,444	*n* = 7,596	0.589	*n* = 3,924	*n* = 247	0.192
	0.5%	0.5%		1.4 %	2.4 %	
**Domestic violence**	*n* = 4,444	*n* = 7,266	< 0.001	*n* = 3,924	*n* = 240	0.378
	7.5%	11.4%		10.3%	12.1%	
**Parental separation**	*n* = 4,444	*n* = 8,664	0.012	*n* = 3,924	*n *= 398	0.697
	26.3%	24.3%		33.21%	34.1%	
**Separation from parents**	*n* = 4,444	*n* = 7,605	< 0.001	*n* = 3,924	*n* = 248	< 0.001
	24.3%	20.5%		8.4%	15.3%	
**Maternal mental health problems**	*n* = 4,444	*n* = 5,798	< 0.001	*n* = 3,924	*n* = 479	0.174
	8.4%	11,0%		39.8%	43.01%	
**Parental alcohol or drug problems**	*n* = 4,444	*n* = 9,096	0.839	‐	‐	‐
	9.6%	9.7%		‐	‐	‐
**Physical neglect**	‐	‐	‐	*n* = 3,924	*n* = 223	0.909
	‐	‐	‐	4.8%	4.9%	
**Emotional neglect**	‐	‐	‐	*n* = 3,924	*n* = 255	0.580
	‐	‐	‐	20.1%	21.6%	
**Parental death**	‐	‐	‐	*n* = 3,924	*n* = 601	0.005
	‐	‐	‐	7.2%	10.5%	
**ACE score**			0.002			0.978
**0**	50.4%	49.2%		39.6%	39.8%	
**1**	33.2%	31.4%		28.0%	28.2%	
**2**	12.0%	13.8%		13.6%	14.3%	
**3+**	4.4%	5.5%		18.8%	17.7%	
***Adiposity measures***						
**BMI at 15 y (kg/m^2^)**	*n* = 3,874	*n* = 1,279	0.005	*n* = 3,830	*n* = 273	0.533
**Mean (SD)**	21.35 (3.46)	21.68 (3.82)		21.46 (3.99)	21.62 (3.66)	
**WC at 15 y (cm)**	*n* = 3,219	*n * = 1,043	0.315	*n* = 3,829	*n* = 273	0.137
**Mean (SD)**	76.55 (8.74)	76.87 (9.42)		70.60 (8.82)	71.42 (8.28)	
**BMI at 18 y (kg/m^2^)**	*n* = 3,542	*n* = 1,195	< 0.001	*n* = 3,581	*n* = 392	0.645
**Mean (SD)**	22.71 (4.07)	23.18 (4.56)		23.45 (4.55)	23.34 (4.11)	
**FMI at 18 y (kg/m^2^)**	*n* = 3,418	*n* = 1,117	0.005	*n* = 3,471	*n* = 381	0.001
**Mean (SD)**	6.25 (3.64)	6.62 (4.03)		6.40 (3.90)	5.73 (3.65)	
**Android fat at 18 y (%)**	*n* = 3,433	*n* = 1,122	0.228	*n* = 3,475	*n* = 381	< 0.001
**Mean (SD)**	6.92 (1.32)	6.98 (1.38)		7.86 (1.42)	8.15 (1.44)	

a χ^2^ test for difference between participants with complete data and participants with missing data or lost to follow‐up.

ACE, adverse childhood experience; FMI, fat mass index; WC, waist circumference.

In both cohorts, most of the ACEs as well as the ACE score were inversely associated with SES (Supporting Information Table S4). Lower income was associated with a higher BMI at ages 15 and 18, FMI, and android fat in ALSPAC, whereas family income was positively associated with males' BMI at ages 15 and 18 years, males' WC, and android fat in the 1993 Pelotas Cohort.

### ALSPAC

The ACE most frequently observed was parental separation (26.3%), followed by separation from parents (24.3%). Roughly 50% of the adolescents experienced none of the ACEs, and 4.4% experienced three or more ACEs (Table 2). The prevalence of all ACEs and the ACE score was similar in males and females, except for parental alcohol and drug problems, which was higher in females (Supporting Information Table S5).

In the unadjusted analysis, domestic violence was associated with higher BMI and WC at 15 years, and parental separation was associated with higher android fat at 18 years (Table 3). After adjustment for confounders (Table 4), the association between domestic violence and higher BMI (0.42 kg/m^2^; 95% CI: 0.04‐0.80) and WC (1.97 cm; 95% CI: 0.86‐3.09) at 15 years remained, and an association between physical abuse and higher WC by 1.84 cm (95% CI: 0.46‐3.23) emerged.

**Table 3:** Unadjusted analysis of the association between ACEs and adiposity measures in adolescents: ALSPAC study, United Kingdom, and 1993 Pelotas Cohort, Brazil

	BMI at 15 y (kg/m^2^)	WC at 15 y (cm)	BMI at 18 y (kg/m^2^)	FMI at 18 y (kg/m^2^)	Android fat at 18 y (%)
**ALSPAC, *N* = 4,444**					
**Physical abuse**	0.01 (−0.49 to 0.51)	1.32 (−0.12 to 2.75)	−0.27 (−0.88 to 0.34)	−0.22 (−0.98 to 0.53)	−0.05 (−0.26 to 0.17)
**Sexual abuse**	0.60 (−0.90 to 2.11)	1.13 (−3.46 to 5.72)	0.16 (−1.70 to 2.02)	0.86 (−1.34 to 3.06)	0.10 (−0.55 to 0.75)
**Domestic violence**	0.47 (0.07 to 0.88)	1.75 (0.61 to 2.91)	0.26 (−0.22 to 0.74)	0.48 (−0.13 to 1.10)	0.09 (−0.08 to 0.27)
**Parental separation**	0.19 (−0.05 to 0.44)	0.56 (−0.12 to 1.24)	0.14 (−0.14 to 0.42)	0.23 (−0.06 to 0.51)	0.11 (0.01 to 0.22)
**Separation from parents**	0.07 (−0.18 to 0.32)	0.33 (−0.37 to 1.03)	0.05 (−0.24 to 0.35)	−0.16 (−0.51 to 0.19)	−0.02 (−0.12 to 0.09)
**Maternal mental health problem**	0.28 (−0.11 to 0.67)	0.82 (−0.27 to 1.91)	0.20 (−0.25 to 0.65)	0.24 (−0.30 to 0.78)	0.08 (−0.08 to 0.24)
**ACE score**	*P* = 0.022^a^	*P* = 0.003^a^	*P* = 0.507	*P* = 0.628	*P* = 0.383
**0**	0 (Ref)	0 (Ref)	0 (Ref)	0 (Ref)	0 (Ref)
**1**	0.20 (−0.04 to 0.44)	0.28 (−0.39 to 0.96)	0.18 (−0.10 to 0.46)	−0.00 (−0.34 to 0.34)	0.08 (−0.02 to 0.17)
**2**	0.33 (−0.01 to 0.67)	0.68 (−0.29 to 1.64)	0.24 (−0.16 to 0.65)	0.31 (−0.18 to 0.80)	0.07 (−0.07 to 0.21)
**3+**	0.35 (−0.18 to 0.88)	2.54 (1.06 to 4.02)	0.07 (−0.56 to 0.71)	0.16 (−0.64 to 0.96)	0.11 (−0.11 to 0.34)
**1993 Pelotas Cohort, *N* = 3,924**					
**Physical abuse**	−0.10 (−0.59 to 0.39)	−0.61 (−1.70 to 0.47)	0.09 (−0.47 to 0.66)	0.11 (−0.69 to 0.90)	−0.06 (−0.24 to 0.13)
**Sexual abuse**	−0.84 (−3.62 to 1.93)	−3.28 (−5.68 to −0.88)	−0.47 (−1.69 to 0.76)	0.51 (−1.20 to 2.21)	−0.12 (−0.45 to 0.28)
**Domestic violence**	−0.03 (−0.44 to 0.38)	−0.48 (−1.39 to 0.43)	−0.18 (−0.65 to 0.29)	0.15 (−0.52 to 0.82)	−0.06 (−0.21 to 0.09)
**Parental separation**	−0.18 (−0.44 to 0.09)	−0.45 (−1.04 to 0.14)	−0.15 (−0.46 to 0.15)	−0.23 (−0.67 to 0.20)	−0.07 (−0.17 to 0.03)
**Separation from parents**	−0.24 (−0.69 to 0.21)	−0.67 (−1.67 to 0.33)	−0.04 (−0.57 to 0.49)	0.09 (−0.94 to 0.21)	0.02 (−0.15 to 0.19)
**Maternal mental health problem** a	−0.23 (−0.49 to 0.02)	−0.38 (−0.94 to 0.19)	0.00 (−0.29 to 0.29)	−0.08 (−0.51 to 0.34)	0.06 (−0.03 to 0.16)
**ACE score**	*P* = 0.041^a^	*P* = 0.012^a^	*P* = 0.801	*P* = 0.975	*P* = 0.780
**0**	0 (Ref)	0 (Ref)	0 (Ref)	0 (Ref)	0 (Ref)
**1**	−0.04 (−0.33 to 0.25)	−0.19 (−0.83 to 0.45)	0.05 (−0.28 to 0.39)	−0.09 (−0.58 to 0.40)	0.03 (−0.07 to 0.14)
**2**	−0.24 (−0.60 to 0.12)	−0.51 (−1.31 to 0.30)	−0.08 (−0.49 to 0.34)	−0.08 (−0.67 to 0.51)	−0.02 (−0.16 to 0.11)
**3+**	−0.48 (−0.97 to 0.01)	−1.46 (−2.55 to −0.37)	−0.21 (−0.78 to 0.35)	−0.15 (−0.96 to 0.65)	−0.04 (−0.22 to 0.14)

Coefficients are mean differences in outcome comparing exposed group with unexposed group or comparing each category of ACE score with people experiencing no ACEs.

a Wald test for linear trend; other *P* values correspond to Wald test for heterogeneity.

ACE, adverse childhood experience; FMI, fat mass index; WC, waist circumference.

The higher the ACE score, the higher the BMI and WC at 15 years in unadjusted (Table 3) and adjusted analyses (Table 4); experiencing three or more ACEs was associated with a higher WC by 2.99 cm (95% CI: 1.56‐4.43). No association was found between the ACE score and adiposity measures at age 18. When parental alcohol or drug problems were also included in the score, the result for WC was still evident in both unadjusted (Supporting Information Table S6) and adjusted analyses (Table 5).

**Table 4:** Adjusted analysis of the association between ACEs and adiposity measures in adolescents: ALSPAC study, United Kingdom, and 1993 Pelotas Cohort, Brazil

	BMI at 15 y (kg/m^2^)	WC at 15 y (cm)	BMI at 18 y (kg/m^2^)	FMI at 18 y (kg/m^2^)	Android fat at 18 y (%)
**ALSPAC, *N* = 4,444**					
**Physical abuse**	0.20 (−0.26 to 0.67)	1.84 (0.46‐3.23)	−0.05 (−0.63 to 0.53)	−0.05 (−0.66 to 0.75)	−0.05 (−0.25 to 0.16)
**Sexual abuse**	−0.45 (−1.85 to 0.96)	−0.39 (−4.80 to 4.01)	−0.97 (−2.78 to 0.84)	−0.66 (−2.72 to 1.41)	0.02 (−0.60 to 0.65)
**Domestic violence**	0.42 (0.04 to 0.80)	1.97 (0.86 to 3.09)	0.22 (−0.24 to 0.67)	0.29 (−0.29 to 0.87)	0.07 (−0.09 to 0.24)
**Parental separation**	0.09 (−0.14 to 0.32)	0.52 (−0.16 to 1.19)	0.04 (−0.24 to 0.31)	−0.02 (−0.35 to 0.31)	0.10 (−0.00 to 0.20)
**Separation from parents**	0.08 (−0.15 to 0.31)	0.46 (−0.22 to 1.14)	0.07 (−0.21 to 0.34)	−0.09 (−0.42 to 0.23)	−0.02 (−0.12 to 0.08)
**Maternal mental health problem**	0.25 (−0.12 to 0.61)	0.86 (−0.19 to 1.92)	0.17 (−0.25 to 0.59)	0.06 (−0.43 to 0.46)	0.07 (−0.09 to 0.23)
**ACE score**	*P* = 0.049^a^	*P* = 0.001^a^	*P* = 0.706	*P* = 0.923	*P* = 0.555
**0**	0 (Ref)	0 (Ref)	0 (Ref)	0 (Ref)	0 (Ref)
**1**	0.17 (−0.06 to 0.39)	0.34 (−0.31 to 0.99)	0.14 (−0.12 to 0.40)	−0.06 (−0.37 to 0.26)	0.06 (−0.03 to 0.16)
**2**	0.24 (−0.08 to 0.56)	0.62 (−0.32 to 1.56)	0.16 (−0.22 to 0.54)	0.11 (−0.37 to 0.56)	0.05 (−0.09 to 0.19)
**3+**	0.32 (−0.17 to 0.81)	2.99 (1.56 to 4.43)	0.05 (−0.55 to 0.66)	−0.04 (−0.80 to 0.72)	0.10 (−0.12 to 0.32)
**1993 Pelotas Cohort, *N* = 3,924**					
**Physical abuse**	−0.10 (−0.57 to 0.36)	−0.22 (−1.24 to 0.88)	0.08 (−0.46 to 0.62)	−0.23 (−0.97 to 0.52)	0.01 (−0.17 to 0.19)
**Sexual abuse**	−0.71 (−1.73 to 0.31)	−1.66 (−3.92 to 0.61)	−0.40 (−1.57 to 0.78)	−0.48 (−2.08 to 1.11)	0.10 (−0.29 to 0.49)
**Domestic violence**	0.02 (−0.36 to 0.41)	0.16 (−0.69 to 1.02)	−0.15 (−0.60 to 0.31)	−0.22 (−0.86 to 0.41)	0.01 (−0.14 to 0.16)
**Parental separation**	−0.10 (−0.35 to 0.16)	−0.22 (−0.78 to 0.33)	−0.11 (−0.40 to 0.19)	−0.18 (−0.59 to 0.22)	−0.03 (−0.13 to 0.06)
**Separation from parents**	−0.21 (−0.64 to 0.22)	−0.32 (−1.27 to 0.62)	−0.03 (−0.54 to 0.49)	0.11 (−0.80 to 0.59)	0.05 (−0.12 to 0.21)
**Maternal mental health problem**	−0.16 (−0.40 to 0.09)	−0.25 (−0.79 to 0.29)	0.02 (−0.26 to 0.31)	0.10 (−0.30 to 0.51)	0.07 (−0.02 to 0.17)
**ACE score**	*P* = 0.163^a^	*P* = 0.259^a^	*P* = 0.661^a^	*P* = 0.543^a^	*P* = 0.078
**0**	0 (Ref)	0 (Ref)	0 (Ref)	0 (Ref)	0 (Ref)
**1**	0.02 (−0.25 to 0.30)	−0.12 (−0.72 to 0.49)	0.08 (−0.24 to 0.40)	0.10 (−0.37 to 0.57)	0.04 (−0.06 to 0.14)
**2**	−0.13 (−0.48 to 0.21)	−0.18 (−0.94 to 0.57)	−0.03 (−0.34 to 0.37)	0.02 (−0.54 to 0.58)	0.03 (−0.10 to 0.17)
**3+**	−0.34 (−0.81 to 0.12)	−0.66 (−1.69 to 0.37)	−0.14 (−0.69 to 0.41)	−0.38 (−1.14 to 0.39)	0.05 (−0.13 to 0.23)

Coefficients are mean differences in outcome comparing exposed group with unexposed group or comparing each category of ACE score with people experiencing no ACEs.

Adjusted for family income, maternal education/schooling, maternal age, maternal smoking at pregnancy, maternal prepregnancy BMI, birth weight, skin color, and gender.

Wald test for linear trend; other *P* values correspond to Wald test for heterogeneity.

ACE, adverse childhood experience; FMI, fat mass index; WC, waist circumference.

**Table 5:** Adjusted sensitivity analysis of the association between ACE score in each cohort and adiposity measures in adolescents and with the additional ACEs: ALSPAC study, United Kingdom, and 1993 Pelotas Cohort, Brazil

	BMI at 15 y (kg/m^2^)	WC at 15 y (cm)	BMI at 18 y (kg/m^2^)	FMI at 18 y (kg/m^2^)	Android fat at 18 y (%)
**ALSPAC, *N* = 4,444**					
**Parental alcohol or drug problem**	−0.21 (−0.55 to 0.12)	−0.05 (−1.05 to 0.96)	−0.42 (−0.81 to −0.02)	−0.37 (−0.83 to 0.09)	0.06 (−0.08 to 0.21)
**ACE score** a	*P* = 0.477	*P* = 0.002^c^	*P* = 0.729	*P* = 0.869	*P* = 0.198
**0**	0 (Ref)	0 (Ref)	0 (Ref)	0 (Ref)	0 (Ref)
**1**	0.09 (−0.13 to 0.32)	0.09 (−0.57 to 0.75)	0.12 (−0.15 to 0.38)	−0.09 (−0.41 to 0.23)	0.09 (−0.00 to 0.19)
**2**	0.23 (−0.07 to 0.53)	0.76 (−0.14 to 1.66)	0.11 (−0.25 to 0.48)	0.06 (−0.38 to 0.49)	−0.04 (−0.10 to 0.17)
**3+**	0.16 (−0.27 to 0.58)	2.09 (0.86 to 3.32)	−0.11 (−0.61 to 0.41)	−0.16 (−0.81 to 0.49)	0.13 (−0.05 to 0.32)
**1993 Pelotas Cohort, *N* = 3,924**					
**Physical neglect**	−0.58 (−1.14 to −0.02)	−1.05 (−2.28 to 0.18)	−0.61 (−1.27 to 0.04)	−0.77 (−1.69 to 0.15)	−0.18 (−0.41 to 0.05)
**Emotional neglect**	0.14 (−0.16 to 0.44)	0.15 (−0.51 to 0.81)	0.23 (−0.12 to 0.58)	0.17 (−0.32 to 0.65)	0.06 (−0.05 to 0.18)
**Parental death**	−0.16 (−0.63 to 0.29)	−0.54 (−1.56 to 0.47)	0.09 (−0.44 to 0.63)	−0.21 (−0.95 to 0.53)	−0.01 (−0.19 to 0.17)
**ACE score** b	*P* = 0.243^c^	*P* = 0.509	*P* = 0.672	*P* = 0.508^c^	*P* = 0.358
**0**	0 (Ref)	0 (Ref)	0 (Ref)	0 (Ref)	0 (Ref)
**1**	0.05 (−0.25 to 0.35)	−0.09 (−0.74 to 0.57)	0.18 (−0.16 to 0.53)	0.15 (−0.36 to 0.66)	0.09 (−0.03 to 0.20)
**2**	−0.06 (−0.41 to 0.28)	−0.05 (−0.80 to 0.71)	−0.01 (−0.39 to 0.42)	−0.04 (−0.61 to 0.53)	0.10 (−0.03 to 0.23)
**3+**	−0.22 (−0.59 to 0.16)	−0.60 (−1.43 to 0.23)	−0.02 (−0.46 to 0.42)	−0.19 (−0.80 to 0.43)	0.03 (−0.12 to 0.18)

Coefficients are mean differences in outcome comparing exposed group with unexposed group or comparing each category of ACE score with people experiencing no ACEs.

Adjusted for family income, maternal education/schooling, maternal age, maternal smoking at pregnancy, maternal prepregnancy BMI, birth weight, skin color, and gender.

a ACE score in ALSPAC: physical abuse, sexual abuse, domestic violence, parental separation, separation from parents, maternal mental health problems, and parental alcohol or drug problem.

b ACE score in the 1993 Pelotas Cohort: physical abuse, sexual abuse, domestic violence, parental separation, separation from parents, maternal mental health problems, parental death, physical neglect, and emotional neglect.

c Wald test for linear trend; other *P* values correspond to Wald test for heterogeneity.

ACE, adverse childhood experience; FMI, fat mass index; WC, waist circumference.

#### Gender‐stratified analysis

In the unadjusted analysis, in addition to the association observed between domestic violence and both higher BMI and WC, physical abuse was also associated with higher WC at 15 years in males (Supporting Information Table S7). In females (Supporting Information Table S8), only parental separation was associated with higher android fat percentage (0.19%; 95% CI: 0.06‐0.31). After adjustment for confounders, the associations observed in the unadjusted analysis were still evident in males (Supporting Information Table S8) and females (Supporting Information Table S9).

The association between higher ACE score and both higher BMI and WC at 15 years was noticeable in males (Supporting Information Table S9) but not females (Supporting Information Table S10), and the results did not change when parental alcohol or drug problems were included in the score (Supporting Information Tables S11‐S12).

### 1993 Pelotas Cohort

Maternal mental health problems was the ACE with the highest prevalence (39.8%), followed by parental separation (33.2%) (Table 2). Female adolescents were more likely to report physical abuse, sexual abuse, domestic violence, separation from parents, and emotional neglect as well as a higher number of ACEs (Supporting Information Table S5).

In the unadjusted analysis, only sexual abuse was associated with lower WC at 15 years (Table 3). After adjustment for confounders, this association was no longer apparent (Table 4). From the additional ACEs available in the 1993 Pelotas Cohort, physical neglect was associated with a lower BMI by 0.58 kg/m^2^ (95% CI: −1.14 to −0.02) at age 15 (Table 5).

The ACE score was inversely associated with BMI and WC at 15 years in the unadjusted (Table 3) but not adjusted analysis (Table 4). Similar results for the ACE score were observed in both the unadjusted (Supporting Information Table S6) and adjusted analysis (Table 5) when adding physical neglect, emotional neglect, and parental death.

#### Gender‐stratified analysis

In the unadjusted analysis, parental separation was associated with a lower BMI and lower WC at 15 years in males (Supporting Information Table S7), and maternal mental health problems was associated with higher android fat percentage in females (Supporting Information Table S8). After adjustment for confounders, these associations were no longer apparent (Supporting Information Tables S9‐S10); however, an association between separation from parents and a lower BMI at both 15 years (−0.66 kg/m^2^; 95% CI: −1.32 to −0.01) and 18 years (−0.74 kg/m^2^; 95% CI: −1.46 to −0.03) was seen in males (Supporting Information Table S9).

For the ACE score, no association was observed in either unadjusted (Supporting Information Tables S7‐S8) or adjusted analyses when stratified by gender (Supporting Information Tables S9‐S10), and the same was found when the additional ACEs were added to the score (Supporting Information Tables S11‐S12).

## Discussion

This study described the association between ACEs and adiposity measures in adolescents in two cohorts with different socioeconomic and cultural profiles. Some associations were found between the exposure to ACEs and adiposity measures in adolescence (mainly 15 years), with positive associations in the United Kingdom cohort and negative associations in the Brazilian cohort; however, most associations were null, and the associations that were observed were not consistent across cohorts and were generally stronger in males.

The higher prevalence of ACEs found in Brazil might be partially explained by sociocultural and environmental factors, which can affect not only the occurrence of ACEs but also the perception and reporting of adversities 15. In this study, however, besides these factors, a great part of the variation in the prevalence could be due to the different reporting sources of ACEs and the recall period in the two cohorts. In the 1993 Pelotas Cohort, the ACEs were self‐reported in adolescence, while in ALSPAC, they were reported by the mother, which could potentially underestimate the prevalence of ACEs not “visible” to outsiders or in which the mother or father was the perpetrator 16. In the 1993 Pelotas Cohort, most of the ACEs were assessed at 15 years, while in ALSPAC, they were assessed mainly up to 9 or 11 years. There is evidence showing that ACEs are more likely to happen either in early childhood or after the onset of puberty 17, so the recall period in ALSPAC may have had some influence on the prevalence. Gender differences in the prevalence of ACEs observed in Brazil have also been found in other studies in which females had a higher occurrence of adversities 1, 16, 18.

In our study, there was some evidence of associations of domestic violence, physical abuse, and cumulative exposure to ACEs with increased adiposity at 15 years in ALSPAC but not at 18 years. However, in the 1993 Pelotas Cohort, the few associations that were seen were negative. In contrast to some 19, 20 but not all studies 21, 22, in our study, when the association between ACEs and adiposity measures differed by gender, it was stronger for males. Several mechanisms are potentially involved in the association between ACEs and adiposity. The stress experienced in early life may induce changes in the ability of the hypothalamic‐pituitary‐adrenal axis to respond to stress 23, 24. This could lead to excessive cortisol levels, which may influence the reward system contributing to increased food intake 23, and lipolysis inhibition, which can contribute to fat (principally visceral fat) accumulation 23, 24. Studies have shown that hypothalamic‐pituitary‐adrenal axis functioning differs in adolescence and adulthood, with age and pubertal development as well as gender differences influencing its activity 25, 26. This could possibly explain the lack of association with adiposity measures at 18 years as well as the gender‐differences observed. Furthermore, it is possible that the effect of ACEs is transient and the association is only observed close to the exposure occurrence. However, some authors have suggested that the relationship between ACEs and higher adiposity has an incubation period 27 and emerges later in life. This has been observed in a study that assessed the association between childhood maltreatment and BMI trajectory from 7 to 50 years 28. In childhood, those who experienced maltreatment had a lower or similar BMI than those who did not; however, the BMI was higher in those who experienced maltreatment in mid‐adulthood (after 45 years) 28.

Our study found a dose‐response association between the ACE score and both BMI and WC at 15 years (but not at 18 years) in ALSPAC, and the association was stronger for males. However, in the 1993 Pelotas Cohort, no association was found with the ACE score. Previous studies carried out in high‐income countries found that the number of ACEs was associated with higher BMI and WC 29, and higher odds of overweight 30 in adolescents and a dose‐response relationship between the accumulation of ACEs and both BMI and WC has also been observed 29.

The different confounding structure observed in the two cohorts might explain the different patterns of association found. In both ALSPAC and the 1993 Pelotas Cohort, SES was inversely related to most ACEs; however, while higher family income was associated with lower adiposity in ALSPAC, adiposity was positively associated or had no association with SES in the 1993 Pelotas Cohort. Therefore, residual confounding by socioeconomic factors could explain the positive relationship between ACEs and adiposity measures in ALSPAC and the lack of associations or opposite direction of associations in the 1993 Pelotas Cohort. It is also possible that the association between ACEs and BMI is context‐specific, with sociocultural factors having a great influence. The way the ACEs are perceived may vary across cultures (e.g., in several societies, minor acts of physical force against a child are seen as an acceptable form of discipline 31), and this could influence the association of these factors with adiposity.

Given that ACEs are likely to be socially patterned, this study used a cross‐cohort comparison to explore the association between ACEs and adiposity measures in two different socioeconomic contexts. In both ALSPAC and the 1993 Pelotas Cohort, the outcomes were assessed at comparable ages by using detailed measures, and it was possible to explore not only general adiposity but also central adiposity. However, some measures were not available in both cohorts to explore the continuity of the association (e.g., WC at 18 years). Furthermore, it was not possible to explore differences in sociocultural factors, such as parenting concerns, parental knowledge and skills in child development and caregiving, parental affection, and parental history of childhood adversities in both cohorts, which could also explain the different prevalence of ACEs and the different patterns of association with adiposity across both cohorts 15, 32.

Even though similar ACEs were available in both cohorts, the questions, timing of assessment, and reporting source were different, which limits the comparison between the studies, especially regarding the prevalence of ACEs. However, the main objective was to compare the existence of an association between ACEs and adiposity measures in both settings.

ACEs tend to co‐occur 6, 33, and for this reason, we used a score of cumulative ACE exposure. Using a simple summed score as we have done in this paper makes the unrealistic assumption that each ACE has the same magnitude and direction of association with the outcome 34. However, we opted to use this approach to facilitate the cross‐cohort comparison. In our analysis of individual ACEs, we did not adjust for other ACEs, as the causal pathways are likely to be complex and uncertain; one could be a consequence of another and, therefore, be a mediator rather than a confounder.

The high rate of dropout and/or incomplete data, especially in ALSPAC, has to be considered. In ALSPAC and the 1993 Pelotas Cohort, similar to other cohorts, missing data and loss to follow‐up were more common in those from socioeconomically deprived backgrounds 35. Furthermore, participants included in the analysis differed concerning the occurrence of ACEs and adiposity measures, especially in ALSPAC. However, the associations between ACEs and adiposity are less likely than prevalence to be affected by bias because of missing data 36. Moreover, multiple imputation was used to minimize selection bias and increase precision in the analysis 37.

## Conclusion

This study showed little evidence for the association between ACEs and adiposity in adolescence, and the results found were not consistent across the two cohorts. Other studies, including those in low‐ and middle‐income countries, are necessary to better understand the relationship between ACEs and adiposity in this age group and how this association differs by gender, as well as to explore whether the association persists or changes in adulthood in different socioeconomic and cultural contexts.

## Supporting information

Supporting Information 1
