# Supplementary material for: Adverse Childhood Experiences (ACEs) and Adiposity in Adolescents: A Cross‐Cohort Comparison
Source: Obesity (Silver Spring). 2017 Nov 14;26(1):150–9. doi: 10.1002/oby.22035 (PMC5765459; doi:10.1002/oby.22035)
Supplement: Supplementary file 1 — Supporting Information 1 [file OBY-26-150-s001.docx]

**Adverse childhood experiences (ACEs) and adiposity in adolescents: a cross-cohort comparison**

Running head: ACEs and adiposity measures in adolescence

Ana Luiza G Soares^1,2^, Alicia Matijasevich^3^, Ana MB Menezes^1^, Maria Cecília Assunção^1^, Fernando C Wehrmeister^1^, Laura D Howe^2*^, Helen Gonçalves^1*^

^1^ Postgraduate Program in Epidemiology, Federal University of Pelotas, Pelotas, Brazil

^2^ MRC Integrative Epidemiology Unit at the University of Bristol, School of Social & Community Medicine, University of Bristol, Bris­tol, UK

^3^ Department of Preventive Medicine, School of Medicine, University of São Paulo, São Paulo, Brazil

* these authors contributed equally to this work

Correspondence concerning this paper should be addressed to:

Name: Ana Luiza G Soares

Mailing address: School of Social and Community Medicine, University of Bristol. Oakfield House, Oakfield Grove. Bristol, UK, BS8 2BN

Email: analuiza.soares@bristol.ac.uk

Phone/ Fax: +44 0117 331 3341

Abbreviations

ACE: adverse childhood experience

ALSPAC: The Avon Longitudinal Study of Parents and Children

BMI: body mass index

FMI: fat mass index

SES: socioeconomic status

WC: waist circumference

**ONLINE SUPPORTING INFORMATION**

*Details of assessment of adiposity measures*

In both cohorts, weight and height were measured at a research clinic visit, and BMI was calculated by dividing weight (kg) by height squared (m^2^). In ALSPAC, height was measured using a Harpenden stadiometer (Holtain, Crymych), and weight was measured using a Tanita TBF 305 body fat analyzer and weighing scale (Tanita). In the 1993 Pelotas Cohort, height was measured using an aluminium stadiometer (C.M.S. weighing equipment Ltd), and weight was measured using an electronic scale connected to the BodPod^®^.

In ALSPAC, WC was measured with a flexible tape at the mid-point between the lower ribs and the iliac crest to the nearest 1mm. In the 1993 Pelotas Cohort, it was measured at the narrowest point in the waist with a flexible tape to the nearest 1mm.

FMI was calculated by dividing total fat mass (kg) by height squared (m^2^). Total body fat mass was obtained in both cohorts from dual-energy x-ray absorptiometry (DXA), using Lunar Prodigy DXA scanner (GE Medical Systems Lunar). DXA scans were not performed in participants who were pregnant or suspected to be pregnant, wheelchair users, individuals with osteoarticular deformities, extremely obese individuals, those with height over 192 cm, and those who had implanted metal pins, screws, plates and non-removable metallic objects (body piercings and/or chains).

Android fat mass was measured at the region automatically defined by the DXA scanner, with the caudal limit at the top of the iliac crest and the upper boundary at the base of the skull. Android fat percentage was calculated as a proportion of android fat mass (kg) to total body fat mass (kg).

*Details of assessment of covariates included in the analyses*

In ALSPAC, family income was measured at 33 and 47 months after delivery, and a mean family income was calculated; within-cohort quintiles of family income were generated from the full available sample. Maternal education in 5 categories (CSE – Certificate of secondary education; Vocational degree; O level – ordinary level; A level – advanced level; University degree) was assessed at 32 weeks of pregnancy. Maternal age (years) was collected at 8 weeks gestation. Maternal smoking was based on maternal self-report during pregnancy (8, 18 and 32 weeks) and 8 weeks after birth, and those mothers who smoked at least one cigarette per day at any trimester of pregnancy were classified as smokers. Birth weight was extracted from routine hospital records. Information on ethnicity was reported at 12 years (white, black/mixed/other).

In the 1993 Pelotas Cohort, family income of the month prior to the delivery (quintiles), maternal schooling (0-4 years; 5-8 years; 9-11 years; 12+ years), maternal age (years), maternal smoking status, maternal pre-pregnancy BMI, and birth weight were assessed at the perinatal visit. Quintiles of family income were generated based on the full original sample. Maternal smoking status was based on maternal self-report, and those mothers who smoked at least one cigarette per day during any time of pregnancy were considered smokers. Birth weight was measured at perinatal visit. Skin color (white, non-white) was assessed at 11-year follow-up visit.

In both cohorts, maternal pre-pregnancy weight and height were self-reported (at 12 weeks of gestation in ALPAC and at the perinatal visit in 1993 Pelotas Cohort), and pre-pregnancy BMI was calculated diving weight (kg) by height squared (m^2^). Maternal pre-pregnancy BMI was categorized into: underweight (< 18.5 kg/m^2^), normal (18.5 – 24.9 kg/m^2^), overweight (25.0 – 29.9 kg/m^2^) and obese (≥ 30 kg/m^2^).

**Table S1.** Distribution of the Characteristics in the Observed and Imputed Data in ALSPAC.

| **Variable** | **Model used** | **N available** | **% imputed** | **Distribution:**  **Mean (SE) for continuous variables, % for categorical variables** | | | |
| --- | --- | --- | --- | --- | --- | --- | --- |
|  |  |  |  | Males | | Females | |
|  |  |  |  | Observed | Imputed | Observed | Imputed |
| Family income | Ologit | 4,106 | 7.6 |  |  |  |  |
| 1^st^ (poorer) |  |  |  | 11.7 | 11.8 | 12.7 | 13.0 |
| 2^nd^ |  |  |  | 17.8 | 17.7 | 18.7 | 18.8 |
| 3^rd^ |  |  |  | 25.6 | 25.7 | 24.1 | 24.1 |
| 4^th^ |  |  |  | 19.2 | 19.2 | 19.6 | 19.6 |
| 5^th^ (better off) |  |  |  | 25.7 | 25.6 | 24.9 | 24.5 |
| Maternal education | Ologit | 4,395 | 1.1 |  |  |  |  |
| CSE/none |  |  |  | 9.0 | 9.1 | 9.9 | 10.1 |
| Vocational |  |  |  | 7.4 | 7.5 | 7.5 | 7.5 |
| O-level |  |  |  | 34.4 | 34.4 | 36.1 | 36.1 |
| A-level |  |  |  | 29.4 | 29.2 | 27.7 | 27.6 |
| Degree |  |  |  | 19.8 | 19.8 | 18.8 | 18.7 |
| Ethnicity | Logit | 3,761 | 15.4 |  |  |  |  |
| White |  |  |  | 95.7 | 95.5 | 95.9 | 95.6 |
| Black/mixed |  |  |  | 4.3 | 4.5 | 4.1 | 4.4 |
| Maternal age (years) | Regress | 4,351 | 2.1 | 29.54 (4.40) | 29.52 (4.41) | 29.21 (4.39) | 29.20 (4.39) |
| Maternal BMI (kg/m^2^) | Regress | 4,142 | 6.8 | 22.91 (3.79) | 22.92 (3.80) | 22.80 (3.53) | 22.81 (3.54) |
| Birth weight (g) | Regress | 4,391 | 1.2 | 3487.98 (564.95) | 3488.00 (564.63) | 3382.48 (489.39) | 3381.50 (489.42) |
| BMI at 15 years (kg/m^2^) | Regress | 3,874 | 12.8 | 20.97 (3.32) | 20.99 (3.34) | 21.69 (3.56) | 21.79 (3.70) |
| WC (cm) | Regress | 3,219 | 27.6 | 76.86 (8.75) | 76.86 (8.75) | 76.29 (8.72) | 76.29 (8.72) |
| BMI at 18 years (kg/m^2^) | Regress | 3,542 | 20.3 | 22.54 (3.84) | 22.63 (3.93) | 22.84 (4.23) | 22.94 (4.26) |
| FMI at 18 years (kg/m^2^) | Regress | 3,433 | 22.7 | 4.31 (3.04) | 4.55 (5.62) | 7.80 (3.33) | 7.91 (3.58) |
| Android fat (%) | Regress | 3,433 | 22.7 | 7.21 (1.37) | 7.21 (1.37) | 6.70 (1.23) | 6.70 (1.23) |

BMI: body mass index; FMI: fat mass index

**Table S2.** Distribution of the Characteristics in the Observed and Imputed Data in 1993 Pelotas Cohort.

| **Variable** | **Model used** | **N available** | **% imputed** | **Distribution:**  **Mean (SE) for continuous variables, % for categorical variables** | | | |
| --- | --- | --- | --- | --- | --- | --- | --- |
|  |  |  |  | Males | | Females | |
|  |  |  |  | Observed | Imputed | Observed | Imputed |
| Family income | Ologit | 3,852 | 1.8 |  |  |  |  |
| 1^st^ (poorer) |  |  |  | 18.7 | 18.8 | 19.3 | 19.3 |
| 2^nd^ |  |  |  | 24.7 | 24.7 | 22.8 | 22.8 |
| 3^rd^ |  |  |  | 17.0 | 17.0 | 17.8 | 17.8 |
| 4^th^ |  |  |  | 20.3 | 20.3 | 20.3 | 20.3 |
| 5^th^ (better off) |  |  |  | 19.3 | 19.3 | 19.8 | 19.8 |
| Maternal education | Ologit | 3,918 | 0.2 |  |  |  |  |
| 0 – 4 |  |  |  | 25.7 | 25.7 | 27.6 | 27.6 |
| 5 – 8 |  |  |  | 49.3 | 49.3 | 47.4 | 47.4 |
| 9 – 11 |  |  |  | 17.1 | 17.1 | 17.9 | 17.9 |
| 12 + |  |  |  | 7.9 | 7.9 | 7.1 | 7.1 |
| Skin color | Logit | 3,913 | 0.3 |  |  |  |  |
| White |  |  |  | 67.8 | 67.7 | 65.3 | 65.3 |
| Black/mixed |  |  |  | 32.2 | 32.3 | 34.7 | 34.7 |
| Maternal BMI (kg/m^2^) | Regress | 3,837 | 2.2 | 22.82 (3.91) | 22.83 (3.91) | 22.89 (3.69) | 22.89 (3.69) |
| Birth weight (g) | Regress | 3,918 | 0.2 | 3245.36 (525.37) | 3245.36 (525.20) | 3115.38 (507.44) | 3115.42 (507.40) |
| BMI at 15 years (kg/m^2^) | Regress | 3,830 | 2.4 | 21.35 (4.03) | 21.35 (4.02) | 21.57 (3.95) | 21.56 (3.93) |
| WC at 15 years (cm) | Regress | 3,829 | 2.4 | 72.38 (9.04) | 72.37 (9.04) | 68.93 (8.28) | 68.92 (8.25) |
| BMI at 18 years (kg/m^2^) | Regress | 3,581 | 8.7 | 23.38 (4.28) | 23.36 (4.29) | 23.52 (4.79) | 23.56 (4.78) |
| FMI at 18 years (kg/m^2^) | Regress | 3,471 | 11.5 | 4.24 (3.05) | 4.79 (7.21) | 8.38 (3.53) | 8.59 (4.51) |
| Android fat at 18 years (%) | Regress | 3,475 | 11.4 | 8.14 (1.50) | 8.16 (1.50) | 7.61 (1.30) | 7.63 (1.30) |

BMI: body mass index; FMI: fat mass index; WC: waist circumference

**Table S3.** Socioeconomic, Demographic, and Health Characteristics of Participants with Complete Data Compared with Participants with Missing Data or Lost to Follow-up. ALSPAC Study, UK, and 1993 Pelotas Cohort, Brazil

|  | **ALSPAC** | | | **1993 Pelotas Cohort** | | |
| --- | --- | --- | --- | --- | --- | --- |
| **Variables** | **Participants included in the analysis**  **%** | **Participants excluded from the analysis**  **%** | **P value^¥^** | **Participants included in the analysis**  **%** | **Participants excluded from the analysis**  **%** | **P value^1^** |
| Gender | N = 4,444 | N = 9.695 | < 0.001 | N = 3,924 | N = 1,325 | < 0.001 |
| Male | 46.4 | 54.1 |  | 48.2 | 54.0 |  |
| Ethnicity | N = 3,761 | N = 3,258 | < 0.001 | N = 3,913 | N = 508 | 0.208 |
| White | 95.8 | 92.3 |  | 66.5 | 69.3 |  |
| Family income (quintile) | N = 4,160 | N = 5,830 | < 0.001 | N = 3,852 | N = 1,285 | 0.005 |
| 1^st^ (lowest) | 12.2 | 26.8 |  | 19.0 | 23.1 |  |
| 2^nd^ | 18.3 | 20.4 |  | 23.7 | 21.9 |  |
| 3^rd^ | 24.8 | 20.7 |  | 17.4 | 17.0 |  |
| 4^th^ | 19.4 | 15.9 |  | 20.3 | 17.2 |  |
| 5^th^ (highest) | 25.3 | 16.2 |  | 19.6 | 20.8 |  |
| Mother’s education | N = 4,395 | N = 8,066 | < 0.001 |  |  |  |
| CSE/none | 9.5 | 26.1 |  |  |  |  |
| Vocational | 7.4 | 11.1 |  |  |  |  |
| O-level | 35.3 | 34.2 |  |  |  |  |
| A-level | 28.5 | 19.2 |  |  |  |  |
| Degree | 19.3 | 9.4 |  |  |  |  |
| Mother’s schooling (years) |  |  |  | N = 3,918 | N = 1,324 | < 0.001 |
| 0 – 4 |  |  |  | 26.7 | 31.9 |  |
| 5 – 8 |  |  |  | 48.3 | 40.2 |  |
| 9 -11 |  |  |  | 17.5 | 17.9 |  |
| 12 + |  |  |  | 7.5 | 10.0 |  |
| Mother’s age at birth (years) | N = 4,351 | N = 7,459 | < 0.001 | N = 3,924 | N = 1,324 | 0.112 |
| < 20 | 1.2 | 5.0 |  | 16.8 | 19.2 |  |
| 20 – 34 | 86.3 | 85.8 |  | 72.3 | 69.6 |  |
| 35+ | 12.5 | 9.2 |  | 10.9 | 11.2 |  |
| Maternal smoking at pregnancy | N = 4,444 | N = 9,419 | < 0.001 | N = 3,924 | N = 1,325 | 0.960 |
| Yes | 15.7 | 33.3 |  | 33.4 | 33.4 |  |
| Maternal pre-pregnancy BMI | N = 4,142 | 7,462 | 0.011 | N = 3,837 | N = 1,261 | 0.028 |
| Underweight | 4.2 | 5.4 |  | 8.6 | 9.8 |  |
| Normal | 76.1 | 73.6 |  | 68.3 | 70.3 |  |
| Overweight | 14.6 | 15.3 |  | 17.9 | 16.3 |  |
| Obese | 5.1 | 5.7 |  | 5.2 | 3.6 |  |
| *Continuous variables* |  |  |  |  |  |  |
| Birth weight (g) | N = 4,391 | N = 9,488 | < 0.001 | N = 3,918 | N = 1,315 | < 0.001 |
| Mean (SD) | 3431.49 (528.41) | 3358.24 (601.55) |  | 3177.98 (520.16) | 3092.76 (624.20) |  |

^1^ Chi-squared test for the difference between participants with complete data and participants with missing data or loss to follow-up

**Table S4.** Unadjusted Association of Family Income with ACEs and Adiposity Measures in ALSPAC and 1993 Pelotas Cohort.

|  | **ALSPAC** | | **1993 Pelotas Cohort** | |  |
| --- | --- | --- | --- | --- | --- |
|  | Effect measure | p-value^1^ | Effect measure | p-value^1^ | |
| **ACEs** *(Odds ratios per income quintile change)* | | | | |  |
| Physical abuse | 0.92 (0.83, 1.02) | 0.133 | 0.91 (0.83, 0.99) | 0.030 | |
| Sexual abuse | 0.69 (0.51, 0.95) | 0.023 | 0.84 (0.70, 1.02) | 0.075 | |
| Domestic violence | 0.78 (0.71, 0.84) | <0.001 | 0.95 (0.88, 1.02) | 0.145 | |
| Parental separation | 0.76 (0.72, 0.80) | <0.001 | 0.91 (0.86, 0.95) | < 0.001 | |
| Separation from parents | 1.09 (1.03, 1.14) | 0.002 | 0.98 (0.90, 1.06) | 0.585 | |
| Maternal mental health | 0.78 (0.72, 0.84) | < 0.001 | 0.83 (0.79, 0.87) | < 0.001 | |
| ACE score |  |  |  |  | |
| 1 | 0.94 (0.89, 0.99) | 0.021 | 0.87 (0.83, 0.92) | < 0.001 | |
| 2 | 0.81 (0.76, 0.87) | < 0.001 | 0.82 (0.77, 0.88) | < 0.001 | |
| 3+ | 0.70 (0.63, 0.79) | < 0.001 | 0.77 (0.70, 0.84) | < 0.001 | |
| **Outcomes** *(mean difference per income quintile change)* | | | | |  |
| BMI at 15 years (kg/m^2^) ^2^ | -0.17 (-0.25, -0.09) | < 0.001 |  |  | |
| Males |  |  | 0.37 (0.25, 0.51) | < 0.001 | |
| Females |  |  | -0.01 (-0.14, 0.11) | 0.820 | |
| WC at 15 years (cm) | -0.21 (-0.44, 0.02) | 0.072 |  |  | |
| Males |  |  | 1.02 (0.73, 1.31) | <0.001 | |
| Females |  |  | -0.07 (-0.33, 0.19) | 0.596 | |
| BMI at 18 years (kg/m^2^) ^2^ | -0.21 (-0.31, -0.11) | <0.001 |  |  | |
| Males |  |  | 0.34 (0.20, 0.48) | < 0.001 | |
| Females |  |  | -0.15 (-0.30, -0.00) | 0.048 | |
| FMI at 18 years (kg/m^2^) ^2^ | -0.31 (-0.43, -0.20) | < 0.001 | 0.12 (-0.03, 0.27) | 0.120 | |
| Android fat (%) ^2^ | -0.04 (-0.07, -0.01) | 0.017 |  |  | |
| Males |  |  | 0.10 (0.05, 0.15) | <0.001 | |
| Females |  |  | -0.03 (-0.08, 0.01) | 0.135 | |

ACE: adverse childhood experience; BMI: body mass index; FMI: fat mass index; WC: waist circumference

^1^ Wald test for the difference among family income quintiles

^2^ p-value <0.001 for effect modification by gender in 1993 Pelotas Cohort in the associations of family income and BMI at 15y and 18y, WC at 15y and android fat at 18y

**Table S5.** Prevalence of ACEs and ACE Score According to Gender in Adolescents in ALSPAC, UK, and the 1993 Pelotas Cohort, Brazil

|  | **ALSPAC** | | | **1993 Pelotas Cohort** | | | |
| --- | --- | --- | --- | --- | --- | --- | --- |
|  | Males  (N=2,063) | Females  (N=2,381) | p-value^1^ | Males  (N=1,890) | Females  (N=2,034) | p-value^1^ |  |
| Physical abuse | 5.1 | 4.5 | 0.319 | 5.7 | 8.3 | 0.002 |  |
| Sexual abuse | 0.3 | 0.7 | 0.131 | 0.5 | 2.3 | < 0.001 |  |
| Domestic violence | 7.0 | 8.0 | 0.190 | 7.8 | 12.6 | < 0.001 |  |
| Parental separation | 25.0 | 27.5 | 0.064 | 32.7 | 33.7 | 0.515 |  |
| Separation from parents | 24.8 | 23.9 | 0.476 | 7.4 | 9.3 | 0.030 |  |
| Maternal mental health problems | 7.9 | 8.9 | 0.231 | 40.8 | 38.8 | 0.212 |  |
| Parental alcohol or drug problems | 8.6 | 10.5 | 0.040 | - | - | - |  |
| Parental death | - | - | - | 7.1 | 7.3 | 0.825 |  |
| Physical neglect | - | - | - | 5.3 | 4.2 | 0.112 |  |
| Emotional neglect | - | - | - | 12.7 | 27.1 | < 0.001 |  |
| ACE score |  |  | 0.262 |  |  | 0.036 |  |
| 0 | 51.0 | 49.8 |  | 36.3 | 35.9 |  |  |
| 1 | 33.4 | 33.0 |  | 40.0 | 35.9 |  |  |
| 2 | 11.4 | 12.5 |  | 17.6 | 18.5 |  |  |
| 3+ | 4.2 | 4.7 |  | 6.1 | 9.7 |  |  |

ACE: adverse childhood experience

^1^ Wald test for the difference between males and females

**Table S6.** Unadjusted Sensitivity Analysis of the Association Between ACE Score in Each Cohort and Adiposity Measures in Adolescents, and with the additional ACEs. ALSPAC Study, UK, and 1993 Pelotas Cohort, Brazil

|  | BMI at 15 years (kg/m^2^) | WC at 15 years (cm) | BMI at 18 years (kg/m^2^) | FMI at 18 years (kg/m^2^) | Android fat at 18 years (%) |
| --- | --- | --- | --- | --- | --- |
| **ALSPAC** |  |  |  |  |  |
| Parental alcohol or drug problem | -14.8 (-0.51, 0.21) | -0.04 (-1.08, 0.99) | -0.40 (-0.82, 0.02) | -0.17 (-0.67, 0.33) | 0.05 (-0.10, 0.20) |
| ACE score^1^ | p = 0.270 | p = 0.025 | p = 0.558 | p = 0.847 | p = 0.099 |
| 0 | 0 (Ref) | 0 (Ref) | 0 (Ref) | 0 (Ref) | 0 (Ref) |
| 1 | 0.16 (-0.08, 0.40) | 0.11 (-0.57, 0.80) | 0.19 (-0.09, 0.47) | 0.01 (-0.34, 0.35) | 0.11 (0.01, 0.21) |
| 2 | 0.27 (-0.06, 0.60) | 0.68 (-0.25, 1.61) | 0.14 (-0.25, 0.52) | 0.17 (-0.30, 0.64) | 0.04 (-0.10, 0.17) |
| 3+ | 0.26 (-0.19, 0.72) | 1.83 (0.56, 3.09) | -0.03 (-0.56, 0.51) | 0.20 (-0.47, 0.87) | 0.16 (-0.03, 0.35) |
| **1993 Pelotas Cohort** |  |  |  |  |  |
| Physical neglect | -0.60 (-1.19, 0.01) | -0.99 (-2.29, 0.32) | -0.53 (-1.21, 0.15) | -1.07 (-2.04, -0.10) | -0.15 (-0.38, 0.08) |
| Emotional neglect | 0.21 (-0.10, 0.52) | -0.60 (-1.29, 0.09) | 0.32 (-0.03, 0.68) | 1.00 (0.49, 1.52) | -0.05 (-0.17, 0.06) |
| Parental death | -0.24 (-0.72, 0.24) | -0.80 (-1.87, 0.27) | 0.05 (-0.50, 0.61) | -0.32 (-1.07, 0.43) | -0.00 (-0.19, 0.18) |
| ACE score ^2^ | p = 0.112^3^ | p = 0.004^3^ | p = 0.901 | p = 0.955 | p = 0.427^3^ |
| 0 | 0 (Ref) | 0 (Ref) | 0 (Ref) | 0 (Ref) | 0 (Ref) |
| 1 | -0.06 (-0.38, 0.25) | -0.34 (-1.04, 0.35) | 0.10 (-0.26, 0.46) | -0.01 (-0.54, 0.53) | 0.07 (-0.05, 0.18) |
| 2 | -0.17 (-0.53, 0.19) | -0.46 (-1.26, 0.34) | -0.04 (-0.46, 0.38) | -0.06 (-0.66, 0.54) | 0.03 (-0.10, 0.17) |
| 3+ | -0.30 (-0.68, 0.09) | -1.36 (-2.22, -0.49) | -0.02 (-0.47, 0.43) | 0.14 (-0.51, 0.78) | -0.07 (-0.22, 0.08) |

ACE: adverse childhood experience; BMI: body mass index; FMI: fat mass index; WC: waist circumference

Coefficients are mean differences in the outcome comparing the exposed group to the unexposed group or comparing each category of the ACE score with people experiencing no ACEs.

^1^ ACE score in ALSPAC: physical abuse, sexual abuse, domestic violence, parental separation, separation from parents, maternal mental health problem, and parental alcohol or drug problem

^2^ ACE score in the 1993 Pelotas Cohort: physical abuse, sexual abuse, domestic violence, parental separation, separation from parents, maternal mental health problem, physical neglect, emotional neglect, and parental death

^3^ Wald test for linear trend; other p-values correspond to Wald test for heterogeneity

**Table S7.** Unadjusted Analysis of the Association Between ACEs and Adiposity Measures in Male Adolescents. ALSPAC Study, UK, and 1993 Pelotas Cohort, Brazil

|  | BMI at 15 years (kg/m^2^) | WC at 15 years (cm) | BMI at 18 years (kg/m^2^) | FMI at 18 years (kg/m^2^) | Android fat at 18 years (%) |
| --- | --- | --- | --- | --- | --- |
| **ALSPAC** |  |  |  |  |  |
| Physical abuse | 0.31 (-0.35, 0.97) | 2.69 (0.53, 4.85) | -0.08 (-0.93, 0.76) | 0.13 (-1.10, 1.37) | 0.08 (-0.24, 0.40) |
| Sexual abuse | 0.92 (-1.80, 3.63) | 16.75 (-0.42, 33.91) | -0.66 (-4.52, 3.20) | -0.70 (-5.65, 4.25) | 1.11 (-0.45, 2.67) |
| Domestic violence | 0.78 (0.20, 1.35) | 2.84 (1.06, 4.63) | 0.56 (-0.14, 1.26) | 0.72 (-0.40, 1.85) | 0.11 (-0.16, 0.39) |
| Parental separation | 0.26 (-0.08, 0.60) | 0.62 (-0.42, 1.65) | 0.06 (-0.34, 0.47) | -0.02 (-0.62, 0.58) | 0.05 (-0.12, 0.21) |
| Separation from parents | 0.12 (-0.21, 0.46) | 0.60 (-0.42, 1.62) | 0.13 (-0.28, 0.54) | -0.11 (-0.70, 0.47) | -0.02 (-0.18, 0.14) |
| Maternal mental health problem | 0.32 (-0.22, 0.86) | 1.53 (-0.12, 3.17) | 0.28 (-0.38, 0.94) | -0.00 (-0.94, 0.94) | 0.15 (-0.12, 0.41) |
| ACE score | p = 0.011^1^ | p = 0.001^1^ | p = 0.248^1^ | p = 0.851 | p = 0.681 |
| 0 | 0 (Ref) | 0 (Ref) | 0 (Ref) | 0 (Ref) | 0 (Ref) |
| 1 | 0.27 (-0.05, 0.60) | 0.55 (-0.44, 1.53) | 0.16 (-0.23, 0.54) | -0.17 (-0.75, 0.40) | 0.01 (-0.14, 0.16) |
| 2 | 0.45 (-0.02, 0.94) | 1.73 (0.26, 3.21) | 0.26 (-0.33, 0.85) | 0.18 (-0.68, 1.04) | -0.01 (-0.24, 0.22) |
| 3+ | 0.64 (-0.10, 1.38) | 3.05 (0.78, 5.31) | 0.35 (-0.57, 1.27) | 0.18 (-1.22, 1.59) | 0.22 (-0.14, 0.58) |
| **1993 Pelotas Cohort** |  |  |  |  |  |
| Physical abuse | -0.28 (-1.07, 0.51) | -0.45 (-2.22, 1.33) | -0.35 (-1.20, 0.50) | -0.50 (-1.97, 0.96) | -0.10 (-0.41, 0.20) |
| Sexual abuse | -0.84 (-3.65, 1.96) | -2.96 (-9.38, 3.46) | 0.10 (-2.75, 2.95) | -0.86 (-5.69, 3.97) | -0.30 (-1.33, 0.74) |
| Domestic violence | 0.04 (-0.64, 0.72) | 0.10 (-1.42, 1.63) | -0.01 (-0.74, 0.72) | -0.39 (-1.66, 0.88) | 0.02 (-0.25, 0.28) |
| Parental separation | -0.40 (-0.79, -0.01) | -1.03 (-1.90, -0.15) | -0.39 (-0.81, 0.03) | -0.58 (-1.31, 0.15) | -0.11 (-0.26, 0.04) |
| Separation from parents | -0.61 (-1.30, 0.09) | -1.31 (-2.88, 0.26) | -0.67 (-1.43, 0.08) | -0.86 (-2.16, 0.43) | -0.08 (-0.35, 0.19) |
| Maternal mental health problem | -0.24 (-0.61, 0.13) | -0.65 (-1.49, 0.18) | -0.11 (-0.50, 0.29) | -0.09 (-0.79, 0.61) | -0.04 (-0.19, 0.10) |
| ACE score | p = 0.085 | p = 0.067 | p = 0.059^1^ | p = 0.108^1^ | p = 0.173^1^ |
| 0 | 0 (Ref) | 0 (Ref) | 0 (Ref) | 0 (Ref) | 0 (Ref) |
| 1 | -0.09 (-0.50, 0.33) | -0.42 (-1.36, 0.51) | -0.03 (-0.48, 0.43) | -0.15 (-0.96, 0.67) | -0.07 (-0.23, 0.09) |
| 2 | -0.60 (-1.13, -0.07) | -1.46 (-2.65, -0.27) | -0.43 (-1.00, 0.14) | -0.51 (-1.52, 0.50) | -0.13 (-0.33, 0.08) |
| 3+ | -0.61 (-1.40, 0.19) | -1.48 (-3.27, 0.31) | -0.68 (-1.53, 0.18) | -1.19 (-2.67, 0.28) | -0.14 (-0.45, 0.17) |

ACE: adverse childhood experience; BMI: body mass index; FMI: fat mass index; WC: waist circumference

Coefficients are mean differences in the outcome comparing the exposed group to the unexposed group or comparing each category of the ACE score with people experiencing no ACEs.

^1^ Wald test for linear trend; other p-values correspond to Wald test for heterogeneity

**Table S8.** Unadjusted Analysis of the Association Between ACEs and Adiposity Measures in Female Adolescents. ALSPAC Study, UK, and 1993 Pelotas Cohort, Brazil

|  | BMI at 15 years (kg/m^2^) | WC at 15 years (cm) | BMI at 18 years (kg/m^2^) | FMI at 18 years (kg/m^2^) | Android fat at 18 years (%) |
| --- | --- | --- | --- | --- | --- |
| **ALSPAC** |  |  |  |  |  |
| Physical abuse | -0.22 (-0.95, 0.51) | 0.24 (-1.69, 2.16) | -0.43 (-1.29, 0.44) | -0.34 (-1.09, 0.40) | -0.23 (-0.51, 0.05) |
| Sexual abuse | 0.28 (-1.56, 2.13) | 0.16 (-4.60, 4.93) | 0.45 (-1.69, 2.58) | 0.77 (-1.08, 2.61) | 0.02 (-0.65, 0.70) |
| Domestic violence | 0.19 (-0.37, 0.75) | 1.03 (-0.47, 2.54) | 0.01 (-0.64, 0.65) | 0.08 (-0.47, 0.63) | 0.09 (-0.12, 0.31) |
| Parental separation | 0.09 (-0.25, 0.43) | 0.56 (-0.35, 1.47) | 0.18 (-0.21, 0.57) | 0.24 (-0.10, 0.58) | 0.19 (0.06, 0.31) |
| Separation from parents | 0.04 (-0.32, 0.39) | 0.06 (-0.91, 1.02) | -0.01 (-0.42, 0.40) | -0.13 (-0.48, 0.23) | -0.02 (-0.15, 0.11) |
| Maternal mental health problem | 0.20 (-0.33, 0.74) | 0.28 (-1.18, 1.75) | 0.12 (-0.49, 0.74) | 0.24 (-0.29, 0.77) | 0.07 (-0.13, 0.26) |
| ACE score | p = 0.832 | p = 0.178 | p = 0.663 | p = 0.698 | p = 0.164 |
| 0 | 0 (Ref) | 0 (Ref) | 0 (Ref) | 0 (Ref) | 0 (Ref) |
| 1 | 0.13 (-0.21, 0.47) | 0.04 (-0.88, 0.97) | 0.20 (-0.19, 0.59) | 0.13 (-0.20, 0.47) | 0.12 (-0.00, 0.24) |
| 2 | 0.18 (-0.29, 0.66) | -0.08 (-1.36, 1.20) | 0.22 (-0.34, 0.77) | 0.25 (-0.23, 0.73) | 0.15 (-0.02, 0.33) |
| 3+ | 0.08 (-0.66, 0.82) | 2.17 (0.21, 4.14) | -0.16 (-1.02, 0.70) | -0.05 (-0.78, 0.68) | 0.05 (-0.23, 0.33) |
| **1993 Pelotas Cohort** |  |  |  |  |  |
| Physical abuse | -0.02 (-0.65, 0.60) | -0.17 (-1.49, 1.15) | 0.35 (-0.41, 1.12) | -0.10 (-0.84, 0.63) | 0.06 (-0.16, 0.28) |
| Sexual abuse | -0.94 (-2.09, 0.21) | -2.03 (-4.46, 0.41) | -0.66 (-2.08, 0.75) | -0.69 (-2.05, 0.67) | 0.12 (-0.30, 0.54) |
| Domestic violence | -0.11 (-0.63, 0.40) | -0.11 (-1.19, 0.98) | -0.32 (-0.95, 0.30) | -0.32 (-0.93, 0.29) | 0.01 (-0.17, 0.19) |
| Parental separation | 0.02 (-0.34, 0.38) | 0.15 (-0.61, 0.92) | 0.06 (-0.38, 0.51) | 0.00 (-0.42, 0.43) | -0.02 (-0.15, 0.10) |
| Separation from parents | 0.01 (-0.58, 0.60) | 0.19 (-1.04, 1.44) | 0.41 (-0.33, 1.15) | 0.38 (-0.35, 1.12) | 0.15 (-0.06, 0.36) |
| Maternal mental health problem | -0.22 (-0.57, 0.14) | -0.25 (-1.00, 0.49) | 0.11 (-0.32, 0.54) | 0.07 (-0.35, 0.49) | 0.14 (0.02, 0.26) |
| ACE score | p = 0.515 | p = 0.426 | p = 0.884 | p = 0.759 | p = 0.386 |
| 0 | 0 (Ref) | 0 (Ref) | 0 (Ref) | 0 (Ref) | 0 (Ref) |
| 1 | 0.01 (-0.40, 0.41) | -0.14 (-0.99, 0.71) | 0.13 (-0.36, 0.62) | 0.14 (-0.33, 0.62) | 0.10 (-0.03, 0.24) |
| 2 | 0.09 (-0.40, 0.58) | 0.44 (-0.60, 1.47) | 0.24 (-0.37, 0.84) | 0.21 (-0.37, 0.78) | 0.09 (-0.08, 0.25) |
| 3+ | -0.41 (-1.03, 0.21) | -0.74 (-2.05, 0.56) | 0.07 (-0.70, 0.84) | -0.17 (-0.91, 0.57) | 0.14 (-0.07, 0.36) |

ACE: adverse childhood experience; BMI: body mass index; FMI: fat mass index; WC: waist circumference

Coefficients are mean differences in the outcome comparing the exposed group to the unexposed group or comparing each category of the ACE score with people experiencing no ACEs.

**Table S9.** Adjusted Analysis of the Association Between ACEs and Adiposity Measures in Male Adolescents. ALSPAC Study, UK, and 1993 Pelotas Cohort, Brazil

|  | BMI at 15 years (kg/m^2^) | WC at 15 years (cm) | BMI at 18 years (kg/m^2^) | FMI at 18 years (kg/m^2^) | Android fat at 18 years (%) |
| --- | --- | --- | --- | --- | --- |
| **ALSPAC** |  |  |  |  |  |
| Physical abuse | 0.53 (-0.09, 1.15) | 3.46 (1.37, 5.55) | 0.16 (-0.65, 0.97) | 0.26 (-0.97, 1.48) | 0.07 (-0.24, 0.38) |
| Sexual abuse | 0.67 (-1.95, 3.30) | 15.89 (-0.71, 32.48) | -0.92 (-4.79, 2.95) | -1.02 (-6.01, 3.97) | 1.16 (-0.38, 2.70) |
| Domestic violence | 0.92 (0.38, 1.46) | 3.62 (1.88, 5.35) | 0.68 (-0.00, 1.36) | 0.76 (-0.37, 1.89) | 0.13 (-0.14, 0.40) |
| Parental separation | 0.20 (-0.12, 0.53) | 0.56 (-0.46, 1.58) | -0.04 (-0.44, 0.36) | -0.20 (-0.81, 0.41) | 0.06 (-0.10, 0.22) |
| Separation from parents | 0.06 (-0.26, 0.38) | 0.53 (-0.46, 1.52) | 0.04 (-0.35, 0.44) | -0.17 (-0.75, 0.40) | -0.03 (-0.19, 0.13) |
| Maternal mental health problem | 0.34 (-0.18, 0.85) | 1.57 (-0.02, 3.16) | 0.28 (-0.34, 0.90) | -0.04 (-0.96, 0.87) | 0.09 (-0.16, 0.35) |
| ACE score | p = 0.009^1^ | p < 0.001^1^ | p = 0.350^1^ | p = 0.811 | p = 0.770 |
| 0 | 0 (Ref) | 0 (Ref) | 0 (Ref) | 0 (Ref) | 0 (Ref) |
| 1 | 0.23 (-0.08, 0.54) | 0.52 (-0.43, 1.48) | 0.08 (-0.28, 0.45) | -0.26 (-0.82, 0.30) | 0.02 (-0.13, 0.17) |
| 2 | 0.38 (-0.07, 0.83) | 1.59 (0.16, 3.02) | 0.15 (-0.41, 0.71) | 0.06 (-0.78, 0.90) | -0.01 (-0.24, 0.22) |
| 3+ | 0.75 (0.05, 1.46) | 3.83 (1.62, 6.04) | 0.40 (-0.49, 1.29) | 0.03 (-1.40, 1.47) | 0.19 (-0.17, 0.54) |
| **1993 Pelotas Cohort** |  |  |  |  |  |
| Physical abuse | -0.32 (-1.06, 0.42) | -0.47 (-2.14, 1.20) | -0.41 (-1.22, 0.39) | -0.56 (-2.00, 0.88) | -0.05 (-0.35, 0.24) |
| Sexual abuse | -0.53 (-3.20, 2.14) | -1.95 (-8.07, 4.16) | 0.39 (-2.33, 3.10) | -0.64 (-5.38, 4.10) | -0.15 (-1.16, 0.86) |
| Domestic violence | 0.13 (-0.50, 0.77) | 0.40 (-1.03, 1.83) | 0.06 (-0.62, 0.75) | -0.28 (-1.53, 0.97) | 0.03 (-0.22, 0.29) |
| Parental separation | -0.23 (-0.60, 0.14) | -0.55 (-1.39, 0.28) | -0.26 (-0.66, 0.14) | -0.41 (-1.13, 0.31) | -0.08 (-0.23, 0.07) |
| Separation from parents | -0.66 (-1.32, -0.01) | -1.37 (-2.85, 0.11) | -0.74 (-1.46, -0.03) | -0.92 (-2.19, 0.34) | -0.10 (-0.36, 0.17) |
| Maternal mental health problem | 0.01 (-0.35, 0.36) | 0.00 (-0.80, 0.80) | 0.13 (-0.26, 0.51) | 0.20 (-0.52, 0.91) | 0.05 (-0.10, 0.20) |
| ACE score | p = 0.283 | p = 0.482 | p = 0.397 | p = 0.365^1^ | p = 0.707^1^ |
| 0 | 0 (Ref) | 0 (Ref) | 0 (Ref) | 0 (Ref) | 0 (Ref) |
| 1 | 0.12 (-0.27, 0.52) | 0.13 (-0.76, 1.02) | 0.16 (-0.27, 0.59) | 0.11 (-0.70, 0.92) | -0.00 (-0.16, 0.16) |
| 2 | -0.33 (-0.83, 0.17) | -0.69 (-1.82, 0.45) | -0.20 (-0.75, 0.34) | -0.21 (-1.22, 0.79) | -0.03 (-0.24, 0.17) |
| 3+ | -0.27 (-1.03, 0.48) | -0.51 (-2.21, 1.20) | -0.39 (-1.22, 0.43) | -0.82 (-2.30, 0.65) | -0.05 (-0.35, 0.26) |

ACE: adverse childhood experience; BMI: body mass index; FMI: fat mass index; WC: waist circumference

Coefficients are mean differences in the outcome comparing the exposed group to the unexposed group or comparing each category of the ACE score with people experiencing no ACEs.

Adjusted for family income, maternal education/schooling, maternal age, maternal smoking at pregnancy, maternal pre-pregnancy BMI, birth weight, skin color and gender

^1^ Wald test for linear trend; other p-values correspond to Wald test for heterogeneity

**Table S10.** Adjusted Analysis of the Association Between ACEs and Adiposity Measures in Female Adolescents. ALSPAC Study, UK, and 1993 Pelotas Cohort, Brazil

|  | BMI at 15 years (kg/m^2^) | WC at 15 years (cm) | BMI at 18 years (kg/m^2^) | FMI at 18 years (kg/m^2^) | Android fat at 18 years (%) |
| --- | --- | --- | --- | --- | --- |
| **ALSPAC** |  |  |  |  |  |
| Physical abuse | -0.05 (-0.73, 0.62) | 0.64 (-1.21, 2.50) | -0.21 (-1.03, 0.62) | -0.15 (-0.86, 0.56) | -0.17 (-0.44, 0.11) |
| Sexual abuse | -1.16 (-2.86, 0.54) | -1.87 (-6.44, 2.70) | -1.14 (-3.12, 0.83) | -0.54 (-2.25, 1.18) | -0.26 (-0.92, 0.40) |
| Domestic violence | 0.00 (-0.53, 0.54) | 0.74 (-0.73, 2.20) | -0.16 (-0.76, 0.45) | -0.07 (-0.59, 0.44) | 0.03 (-0.18, 0.24) |
| Parental separation | -0.02 (-0.34, 0.29) | 0.48 (-0.42, 1.37) | 0.07 (-0.30, 0.45) | 0.13 (-0.20, 0.45) | 0.13 (0.01, 0.26) |
| Separation from parents | 0.11 (-0.22, 0.44) | 0.44 (-0.49, 1.38) | 0.11 (-0.27, 0.49) | -0.01 (-0.34, 0.33) | -0.01 (-0.14, 0.12) |
| Maternal mental health problem | 0.18 (-0.32, 0.69) | 0.34 (-1.07, 1.76) | 0.11 (-0.46, 0.68) | 0.20 (-0.30, 0.70) | 0.04 (-0.15, 0.24) |
| ACE score | p = 0.687^1^ | p = 0.089 | p = 0.614 | p = 0.720 | p = 0.397 |
| 0 | 0 (Ref) | 0 (Ref) | 0 (Ref) | 0 (Ref) | 0 (Ref) |
| 1 | 0.11 (-0.20, 0.43) | 0.19 (-0.70, 1.08) | 0.20 (-0.16, 0.56) | 0.13 (-0.19, 0.44) | 0.10 (-0.02, 0.22) |
| 2 | 0.09 (-0.36, 0.54) | -0.15 (-1.40, 1.10) | 0.17 (-0.35, 0.70) | 0.21 (-0.26, 0.66) | 0.09 (-0.08, 0.27) |
| 3+ | 0.01 (-0.68, 0.69) | 2.39 (0.50, 4.29) | -0.20 (-1.00, 0.60) | -0.10 (-0.78, 0.59) | 0.03 (-0.25, 0.30) |
| **1993 Pelotas Cohort** |  |  |  |  |  |
| Physical abuse | -0.00 (-0.60, 0.59) | -0.16 (-1.43, 1.11) | 0.37 (-0.36, 1.10) | -0.06 (-0.78, 0.65) | 0.05 (-0.16, 0.27) |
| Sexual abuse | -0.82 (-1.91, 0.28) | -1.80 (-4.14, 0.54) | -0.63 (-1.98, 0.73) | -0.55 (-1.88, 0.77) | 0.11 (-0.30, 0.53) |
| Domestic violence | -0.02 (-0.51, 0.47) | 0.05 (-1.00, 1.10) | -0.24 (-0.85, 0.36) | -0.23 (-0.83, 0.37) | 0.00 (-0.17, 0.18) |
| Parental separation | 0.03 (-0.32, 0.39) | 0.11 (-0.64, 0.86) | 0.05 (-0.38, 0.48) | 0.03 (-0.39, 0.45) | 0.00 (-0.13, 0.13) |
| Separation from parents | 0.10 (-0.47, 0.66) | 0.31 (-0.89, 1.52) | 0.49 (-0.22, 1.21) | 0.47 (-0.26, 1.19) | 0.14 (-0.07, 0.35) |
| Maternal mental health problem | -0.26 (-0.60, 0.07) | -0.37 (-1.09, 0.35) | -0.00 (-0.42, 0.42) | 0.06 (-0.35, 0.48) | 0.12 (-0.00, 0.24) |
| ACE score | p = 0.628 | p = 0.471 | p = 0.969 | p = 0.798 | p = 0.424 |
| 0 | 0 (Ref) | 0 (Ref) | 0 (Ref) | 0 (Ref) | 0 (Ref) |
| 1 | -0.02 (-0.41, 0.37) | -0.24 (-1.06, 0.58) | 0.08 (-0.40, 0.55) | 0.14 (-0.32, 0.61) | 0.10 (-0.04, 0.23) |
| 2 | 0.06 (-0.41, 0.53) | 0.32 (-0.69, 1.32) | 0.14 (-0.44, 0.73) | 0.23 (-0.34, 0.80) | 0.10 (-0.07, 0.27) |
| 3+ | -0.36 (-0.95, 0.24) | -0.71 (-1.99, 0.56) | 0.07 (-0.67, 0.82) | -0.08 (-0.81, 0.65) | 0.13 (-0.08, 0.34) |

ACE: adverse childhood experience; BMI: body mass index; FMI: fat mass index; WC: waist circumference

Coefficients are mean differences in the outcome comparing the exposed group to the unexposed group or comparing each category of the ACE score with people experiencing no ACEs.

Adjusted for family income, maternal education/schooling, maternal age, maternal smoking at pregnancy, maternal pre-pregnancy BMI, birth weight, skin color and gender

^1^ Wald test for linear trend; other p-values correspond to Wald test for heterogeneity

**Table S11.** Adjusted Sensitivity Analysis of the Association Between ACE Score in Each Cohort and Adiposity Measures in Male Adolescents, and with the Additional ACEs. ALSPAC Study, UK, and 1993 Pelotas Cohort, Brazil

|  | BMI at 15 years (kg/m^2^) | WC at 15 years (cm) | BMI at 18 years (kg/m^2^) | FMI at 18 years (kg/m^2^) | Android fat at 18 years (%) |
| --- | --- | --- | --- | --- | --- |
| **ALSPAC** |  |  |  |  |  |
| Parental alcohol or drug problem | -0.20 (-0.69, 0.29) | -0.18 (-1.71, 1.36) | -0.52 (-1.13, 0.08) | -0.62 (-1.50, 0.26) | 0.06 (-0.19, 0.31) |
| ACE score^1^ | p = 0.034^3^ | p = 0.001^3^ | p = 0.212^3^ | p = 0.675 | p = 0.700 |
| 0 | 0 (Ref) | 0 (Ref) | 0 (Ref) | 0 (Ref) | 0 (Ref) |
| 1 | 0.09 (-0.22, 0.40) | 0.05 (-0.92, 1.02) | -0.01 (-0.38, 0.37) | -0.36 (-0.93, 0.21) | 0.05 (-0.10, 0.20) |
| 2 | 0.34 (-0.09, 0.76) | 1.61 (0.26, 2.96) | 0.08 (-0.46, 0.61) | -0.05 (-0.84, 0.74) | 0.00 (-0.21, 0.22) |
| 3+ | 0.56 (-0.06, 1.19) | 2.95 (0.99, 4.91) | 0.14 (-0.65, 0.92) | -0.19 (-1.45, 1.06) | 0.17 (-0.14, 0.48) |
| **1993 Pelotas Cohort** |  |  |  |  |  |
| Physical neglect | -0.53 (-1.31, 0.24) | -1.15 (-2.88, 0.59) | -0.79 (-1.64, 0.05) | -1.15 (-2.66, 0.36) | -0.29 (-0.62, 0.03) |
| Emotional neglect | 0.11 (-0.40, 0.62) | 0.21 (-0.95, 1.36) | 0.13 (-0.43, 0.69) | 0.17 (-0.85, 1.18) | 0.05 (-0.16, 0.26) |
| Parental death | -0.50 (-1.17, 0.17) | -1.50 (-3.02, 0.01) | -0.52 (-1.25, 0.21) | -0.95 (-2.25, 0.34) | -0.09 (-0.38, 0.20) |
| ACE score ^2^ | p = 0.134^3^ | p = 0.132^3^ | p = 0.185^3^ | p = 0.226^3^ | p = 0.663 |
| 0 | 0 (Ref) | 0 (Ref) | 0 (Ref) | 0 (Ref) | 0 (Ref) |
| 1 | 0.04 (-0.38, 0.45) | -0.08 (-1.02, 0.86) | 0.15 (-0.30, 0.61) | 0.11 (-0.46, 0.97) | 0.05 (-0.12, 0.22) |
| 2 | -0.12 (-0.61, 0.38) | -0.33 (-1.44, 0.78) | -0.15 (-0.69, 0.39) | -0.40 (-1.39, 0.60) | 0.03 (-0.17, 0.23) |
| 3+ | -0.48 (-1.06, 0.11) | -1.07 (-2.38, 0.25) | -0.40 (-1.04, 0.23) | -0.61 (-1.78, 0.55) | -0.09 (-0.34, 0.15) |

ACE: adverse childhood experience; BMI: body mass index; FMI: fat mass index; WC: waist circumference

Coefficients are mean differences in the outcome comparing the exposed group to the unexposed group or comparing each category of the ACE score with people experiencing no ACEs.

Adjusted for family income, maternal education/schooling, maternal age, maternal smoking at pregnancy, maternal pre-pregnancy BMI, birth weight, skin color and gender

^1^ ACE score in ALSPAC: physical abuse, sexual abuse, domestic violence, parental separation, separation from parents, maternal mental health problem, and parental alcohol or drug problem

^2^ ACE score in the 1993 Pelotas Cohort: physical abuse, sexual abuse, domestic violence, parental separation, separation from parents, maternal mental health problem, physical neglect, emotional neglect, and parental death

^3^ Wald test for linear trend; other p-values correspond to Wald test for heterogeneity

**Table S12.** Adjusted Sensitivity Analysis of the Association Between ACE Score in Each Cohort and Adiposity Measures in Female Adolescents, and with the Additional ACEs. ALSPAC Study, UK, and 1993 Pelotas Cohort, Brazil

|  | BMI at 15 years (kg/m^2^) | WC at 15 years (cm) | BMI at 18 years (kg/m^2^) | FMI at 18 years (kg/m^2^) | Android fat at 18 years (%) |
| --- | --- | --- | --- | --- | --- |
| **ALSPAC** |  |  |  |  |  |
| Parental alcohol or drug problem | -0.25 (-0.71, 0.21) | 0.01 (-1.32, 1.35) | -0.34 (-0.87, 0.20) | -0.15 (-0.62, 0.31) | 0.07 (-0.11, 0.24) |
| ACE score^1^ | p = 0.851 | p = 0.289 | p = 0.458 | p = 0.665 | p = 0.197 |
| 0 | 0 (Ref) | 0 (Ref) | 0 (Ref) | 0 (Ref) | 0 (Ref) |
| 1 | 0.09 (-0.23, 0.41) | 0.14 (-0.77, 1.04) | 0.23 (-0.14, 0.59) | 0.14 (-03.18, 0.46) | 0.13 (0.01, 0.26) |
| 2 | 0.11 (-0.32, 0.54) | -0.00 (-1.22, 1.21) | 0.14 (-0.36, 0.64) | 0.19 (-0.25, 0.64) | 0.06 (-0.10, 0.23) |
| 3+ | -0.12 (-0.70, 0.46) | 1.54 (-0.05, 3.12) | -0.24 (-0.91, 0.43) | -0.10 (-0.68, 0.48) | 0.11 (-0.13, 0.34) |
| **1993 Pelotas Cohort** |  |  |  |  |  |
| Physical neglect | -0.50 (-1.31, 0.32) | -0.60 (-2.34, 1.13) | -0.23 (-1.24, 0.77) | -0.22 (-1.25, 0.81) | 0.01 (-0.30, 0.31) |
| Emotional neglect | 0.12 (-0.25, 0.49) | 0.02 (-0.77, 0.81) | 0.24 (-0.22, 0.70) | 0.15 (-0.61, 0.61) | 0.06 (-0.07, 0.19) |
| Parental death | 0.11 (-0.52, 0.74) | 0.27 (-1.08, 1.61) | 0.63 (-0.14, 1.41) | 0.43 (-0.34, 1.20) | 0.06 (-0.18, 0.30) |
| ACE score ^2^ | p = 0.950 | p = 0.887 | p = 0.722 | p = 0.730 | p = 0.171 |
| 0 | 0 (Ref) | 0 (Ref) | 0 (Ref) | 0 (Ref) | 0 (Ref) |
| 1 | 0.10 (-0.32, 0.52) | -0.01 (-0.91, 0.87) | 0.26 (-0.25, 0.78) | 0.24 (-0.26, 0.75) | 0.14 (-0.00, 0.29) |
| 2 | -0.00 (-0.48, 0.47) | 0.24 (-0.78, 1.26) | 0.17 (-0.42, 0.77) | 0.30 (-0.28, 0.87) | 0.17 (-0.00, 0.34) |
| 3+ | -0.01 (-0.50, 0.49) | -0.21 (-1.26, 0.84) | 0.31 (-0.30, 0.92) | 0.16 (-0.43, 0.76) | 0.13 (-0.04, 0.31) |

ACE: adverse childhood experience; BMI: body mass index; FMI: fat mass index; WC: waist circumference

Coefficients are mean differences in the outcome comparing the exposed group to the unexposed group or comparing each category of the ACE score with people experiencing no ACEs.

Adjusted for family income, maternal education/schooling, maternal age, maternal smoking at pregnancy, maternal pre-pregnancy BMI, birth weight, skin color and gender

^1^ ACE score in ALSPAC: physical abuse, sexual abuse, domestic violence, parental separation, separation from parents, maternal mental health problem, and parental alcohol or drug problem

^2^ ACE score in the 1993 Pelotas Cohort: physical abuse, sexual abuse, domestic violence, parental separation, separation from parents, maternal mental health problem, physical neglect, emotional neglect, and parental death

^3^ Wald test for linear trend; other p-values correspond to Wald test for heterogeneity
